# Recruiting Older Adults in a Medicare Supplement Population for Wellness Intervention Pilot Studies

**DOI:** 10.1093/geroni/igab046.689

**Published:** 2021-12-17

**Authors:** Rachel Ungar, Catherine Zaidel, Rifky Tkatch, Lizi Wu, Laurie Albright, Michael McGinn, Charlotte Yeh

**Affiliations:** 1 UnitedHealth Group, Minnetonka, Minnesota, United States; 2 OptumLabs, Minnetonka, Minnesota, United States; 3 UnitedHealth Group, Oak Park, Michigan, United States; 4 UnitedHealth Group, Minneapolis, Minnesota, United States; 5 AARP Services, Inc., Washington, District of Columbia, United States

## Abstract

Older adults are often underrepresented in the health promotion literature, in part due to challenges in recruiting older adults for such studies. Aging Strong 2020 was specifically designed to address the health needs of older adults. A subset of adults aged 65 and older with an AARP Medicare Supplement plan insured by UnitedHealthcare were recruited for participation in one of eight interventions. Recruitment lists for each program were drawn from a pool previously screened for loneliness, purpose in life, optimism, and resilience, administered by an interactive voice response (IVR) telephone survey. Recruitment efforts were multifaceted and included emails, direct mailers, and phone calls. Incentives ranging from $25-$100 for completing surveys did not correspond with higher recruitment rates. Overall, recruitment phone calls reached 28,058(32%) individuals on the recruitment lists; a total of 1,766 participated, demonstrating that targeted efforts to recruit older adults for research opportunities can be successful.

